# Integration and acceleration of virtual microscopy as the key to successful implementation into the routine diagnostic process

**DOI:** 10.1186/1746-1596-4-3

**Published:** 2009-01-09

**Authors:** Stephan Wienert, Michael Beil, Kai Saeger, Peter Hufnagl, Thomas Schrader

**Affiliations:** 1 VMscope GmbH, Chariteplatz 1, 10117 Berlin, Germany; 2 Lausitz University of Applied Sciences, Großenhainer Str. 57, 01968 Senftenberg, Germany; 3 Institue of Pathology, Charité – University Hospital Berlin, Chariteplatz 1, 10117 Berlin, Germany

## Abstract

**Background:**

The virtual microscopy is widely accepted in Pathology for educational purposes and teleconsultation but is far from the routine use in surgical pathology due to the technical requirements and some limitations. A technical problem is the limited bandwidth of a usual network and the delayed transmission rate and presentation time on the screen.

**Methods:**

In this study the process of secondary diagnostic was evaluated using the "T.Konsult Pathologie" service of the Professional Association of German Pathologists within the German breast cancer screening program. The characteristics of the access to the WSI (Whole Slide Images) have been analyzed to explore the possibilities of prefetching and caching to reduce the presentation and transfer time with the goal to increase user acceptance. The log files of the web server were analyzed to reconstruct the movements of the pathologist on the WSI and to create the observation path. Using a specialized tool the observation paths were extracted automatically from the log files. The attributes linearity, 3-point-linearity, changes per request, and number of consecutive requests were calculated to design, develop and evaluate different caching and prefetching strategies.

**Results:**

The analysis of the observation paths showed that a complete accordance of two image requests is a very rare event. But more frequently a partial covering of two requested image areas can be found. In total 257 diagnostic paths from 131 WSI have been extracted and analysed. On average a diagnostic path consists of 16 image requests and takes 189 seconds between first and last image request. The mean linearity was 0,41 and the mean 3-point-linearity 0,85. Three different caching algorithms have been compared with respect to hit rate and additional image requests on the WSI server. Tests demonstrated that 95% of the diagnostic paths could be loaded without any deletion of entries in the cache (cache size 12,2 Megapixel). If the image parts are stored after JPEG compression this complies with less than 2 MB.

**Discussion:**

WSI telepathology is a technology which offers the possibility to break the limitations of conventional static telepathology. The complete histological slide may be investigated instead of sets of images of lesions sampled by the presenting pathologist. The benefit is demonstrated by the high diagnostic security of 95% accordance between first and second diagnosis.

## Introduction

Virtual microscopy is now widely applied in Pathology. Many studies underline the diagnostic security as well as the versatility of this method [1-5]. The standardization organizations DICOM, HL7 and IHE call Virtual Slides as Whole-Slide-Images (WSI) now [4,6] and integrate the technology into their standardization strategy. In contrast to the increasing application in educations [7-9], the virtual microscopy is far from routine use in surgical pathology. This goes back to several reasons concerning the technical and personal requirements [10,11,15]:

1) Costs: The financial expanses for scanning and storing are currently very high. A slide scanner costs between 60 and 120 TE. A scanned slide has a file size between hundreds of megabytes and several gigabytes. Thus virtual microscopy is used almost by university institutes only.

2) Scanning time: Despite several technical improvements, the scanning time needed for a set of slides does not yet satisfy the requirements of daily routine work. The scanning time lies between 1 and 5 minutes for a biopsy and between 5 and 20 minutes for a surgical specimen

3) Speed of virtual microscopes: With respect to image transfer and display times the working speed of virtual microscopy is lower in comparison to conventional microscopy. Another critical point is the lack of appropriate user interface devices. Conventional computer mouse is inadequate.

Caching and prefetching may speed up image loading, the bottle neck in virtual microscopy. Nevertheless the positive effects of different prefetching and caching technologies depend on the user's behavior. To our knowledge no reports on such user data have been ever published. Nobody knows the diagnostic paths of pathologists exploring histological slides.

From the technical point of view prefetching is useful to load expected areas of a WSI before the user asks for them. Caching holds the information of areas already seen in the fast memory of the computer (cache). The effectiveness depends directly on the behavior of the pathologists. A prefetch is preferred in case of a high probability of forecast the next region of WSI. The caching mechanism is valuable if the user goes several times through the same area or compares current areas with areas already seen.

The telepathological diagnostic process differs between the three application cases primary, secondary and tertiary diagnosis. In case of the primary telepathology the pathologist has to scan all slides and the complete area of each WSI to find the right diagnosis because he or she sees every case the first time and has to work like under the same conditions as in conventional microscopy.

In the secondary diagnostic process the pathologist knows the diagnosis in most cases and his or her task is to confirm the diagnosis. Especially in tumor cases only the confirmation of the tumor entity is necessary. The expected diagnostic process can be faster and it is only necessary to scan the most important slides for the confirmation.

In the tertiary process, cases are discussed in a panel of experts. Concerning the difficulty of the diagnosis every area of a slide is viewed by the pathologists. The diagnostic process needs more time. Details in several areas of the slide may influence the diagnosis significantly. Virtual microscopy offers the possibility to access every part of the slide in highest magnification and best quality.

In this study the process of secondary diagnostic was evaluated using the "T.Konsult Pathologie" service of the Professional Association of German Pathologists within the German breast cancer screening program [16]. The characteristics of the access to the virtual slides was analyzed to explore the possibilities of prefetching and caching to reduce the presentation and transfer time with the goal to increase user acceptance.

## Methods

All individual slides of 149 cases from the routine diagnostic process were scanned with three scanners (Olympus Slide, Zeiss Mirax, Hamamatsu Nanozoomer). 247 WSI were created and stored on the image server. Only biopsies and excisions on standard glass slides with 25 mm × 75 mm × 1 mm were included in this study.

**Figure 1:**
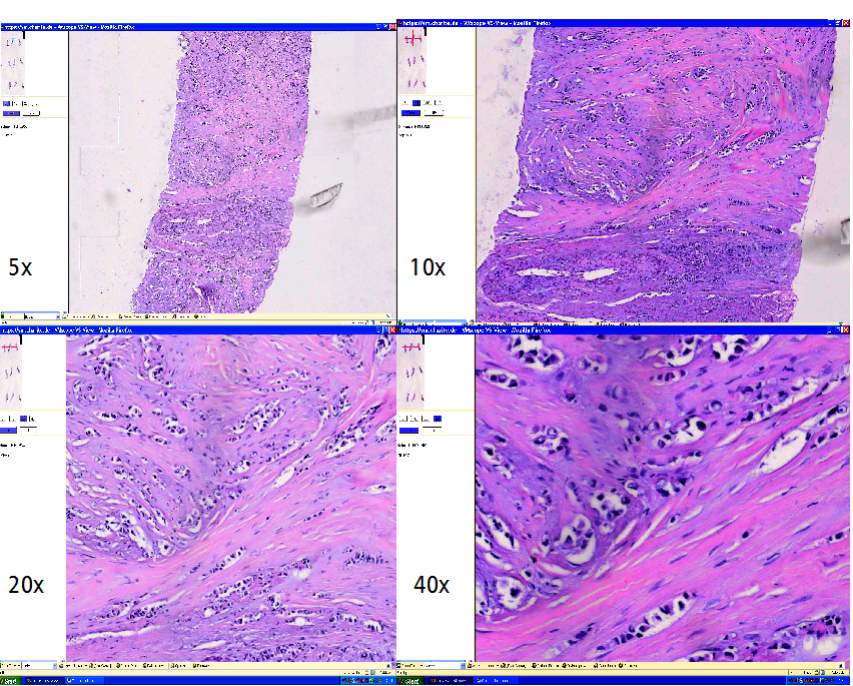
**WSI-Viewer with different magnifications**.

The cases were prepared for the telepathology consultation process in the T.Konsult – Server of the Professional Association of German Pathologists and assigned randomly to 37 pathologists for a confirmation of the primary diagnosis (second opinion). Following clinical data were added to each case for secondary diagnostic process: local case number, gender, age, localization of the lesion, working diagnosis and tumor code expressed in ICD-O. The access to the WSI was realized with "Slide Link List" – a database tool for administration (VMscope GmbH Berlin). After registration and login pathologists reviewed the case information and the WSI's of his or her assigned cases (fig. 1).

The German teleconsultation service T. Konsult Pathologie runs on a server with an Intel Xeon processor, 512 MB RAM and 4 hard drives with 80 GB in a RAID system. Windows 2003 Server system was used as operating system and Microsoft SQL-Server 2000 worked as database engine. Microsoft Internet Information Services operated as web server.

The pathologists were asked to give a second opinion and to classify the cases according the B-Classification. This classification scheme is used to unify diagnoses and to offer comparability. The main decision criterion is the relation to the malignant potential (see table 1).

**Table 1:** The B-Classification and the histologic expression

**Classification**	**Definition**
**B1** **normal**	**Normal or not interpretable tissue** - benign parenchyma with or without minimal changes - artifacts, bleeding
**B2** **Benign**	**Benign Lesion** - fibrocystic changes - fibro adenoma - sclerosing adenosis -ectasia, abscess, fat tissue necrosis - small intraductal papilloma
**B3** **benign, but with increased risk of malignancy or an association with a malignant tumor**	**Lesion with uncertain malignant potential** - large or multiple papillary lesions with or without atypia - scar, complex sclerosing lesion - lobular intraepithelial neoplasia (LIN) - ADH - phylloid tumor or unclear fibroepithelial tumor
**B4** **suspect**	**Suspect for malignancy**
**B5** **malignant**	**Malignant Lesions** **a**) ductal Carcinoma in situ (DCIS) LIN (CLIS) of pleomorphic subtype G3 LIN with comedo necrosis **b**) Carcinoma **c**) status of invasion not decidable **d**) other malignant tumor such as Lymphoma, metastasis or sarcoma

**Figure 2:**
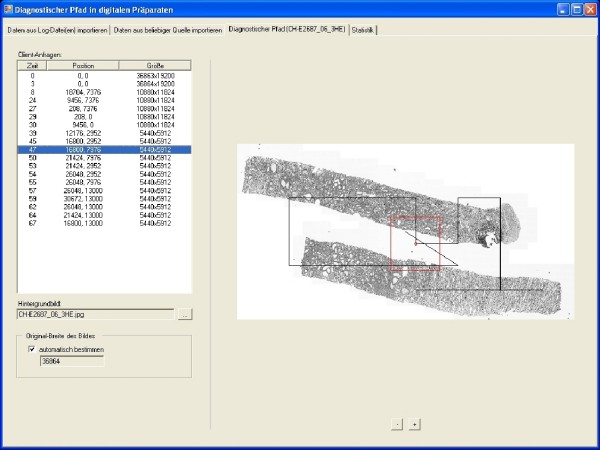
**Demonstration and analysis tool**.

The log files of the web server were analyzed to reconstruct the movements of the pathologist on the WSI and to create the observation path [5]. Using a specialized tool the observation paths were extracted automatically from the log files (fig. 2). The following attributes were calculated

- Linearity – the Euclidean distance between the starting and the end point of the observation path divided by the length of the way between these points

- 3-point-linearity – linearity of all sections of the observation paths, consisting of three following image requests. 

- Changes per request concerning the position in the WSI, the magnification, the size of the requested field of view

- Number of the consecutive requests with the same magnification

**Figure 3:**
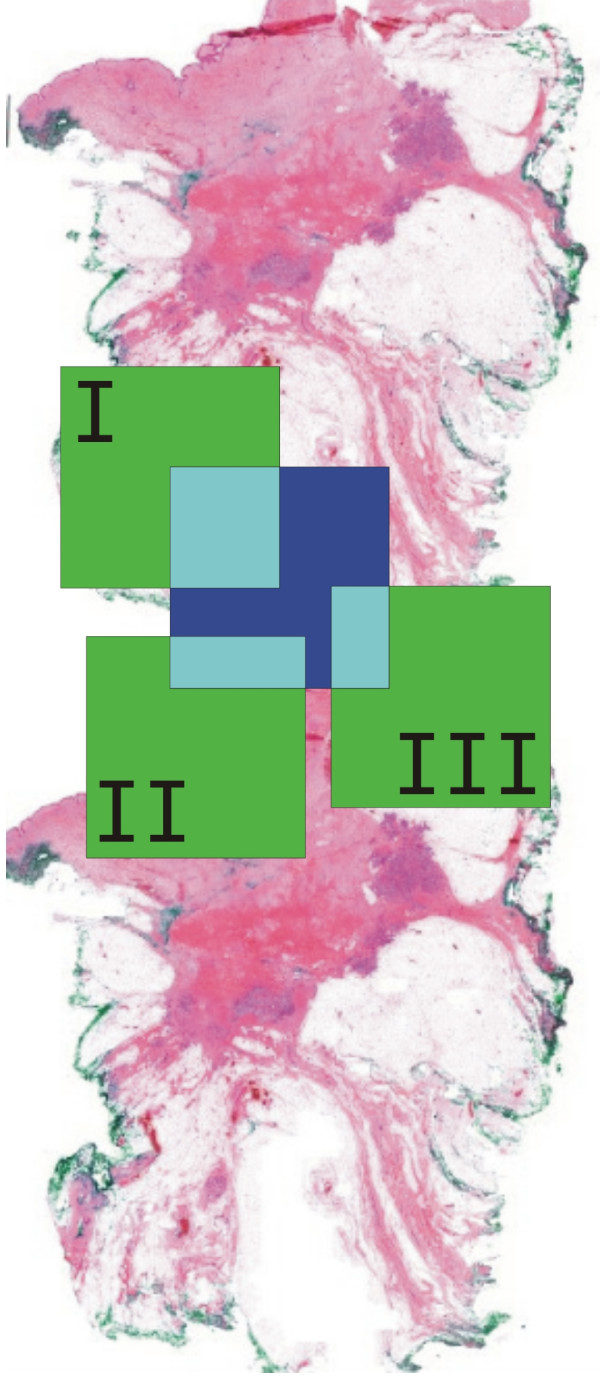
**Caching Algorithms (Green area I, II, III are viewed areas and stored at the cache**. Blue area is the new requested view. Turquoise areas are the overlaps of viewed areas and requested area.) A1 uses the overlaps of I and II, A2 uses only the overlap I and in A3 only the overlaps I and II are used.

The analysis of the observation paths showed that a complete accordance of two image requests is a very rare event. But more frequently a partial covering of two requested image areas can be found. This is the reason for the success of caching algorithms which hold parts of images in the fast memory to load only the missing image parts from the image server. Three different caching algorithms have been developed (fig. 3):

A1: every covering image part in the cache is used

A2: only the largest covering image part in the cache is used

A3: limitation on a certain number of arising parts of images per image request.

The algorithms have been evaluated by their hit rate (amount of pixels from the cache on the number of all requested pixels) and the number of additional requests on the WSI server. Such additional requests appear if only a part of the image request can be satisfied by the cache content. The remaining area must be requested from the WSI server in form of separate rectangles. In this case latency periods and protocol overhead (i.e. HTTP, TCP/IP) of the network is multiple, which is a direct drawback of caching.

**Figure 4:**
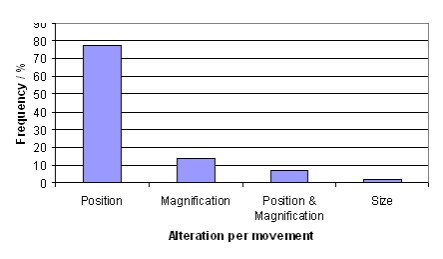
**Linearity**.

As a result of the analysis of the diagnostic paths we found that between two requests often only the position within the WSI is changed but not the resolution (fig 4). The probability of a change of the resolution between two image requests is about 20%. Consequently it makes sense to load an image of identical resolution after two requests with the same resolution have been already placed (prefetch). The linearity of three consecutive image requests was high in the all diagnostic paths which have been analysed. That is why it makes sense to load an image in the same moving direction β and step width as in the image requests before.

## Results

We compared conventional original diagnosis with telepathological diagnosis on the basis of WSI. There was a accordance of 94,56% between conventional and WSI diagnosis with respect to the B-classification. The image quality was good or excellent for 79,84% of the images. The time needed for the diagnostic process of one case was lower than 20 minutes.

**Figure 5:**
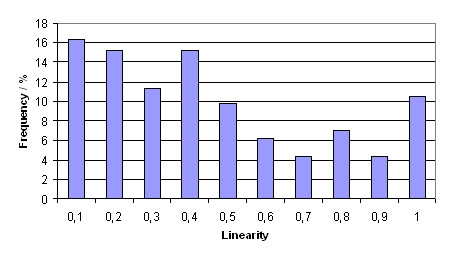
**3-point linearity**.

**Figure 6:**
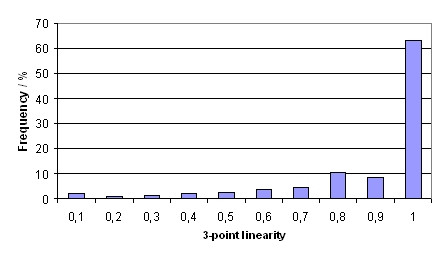
**Alteration per movement**.

**Figure 7:**
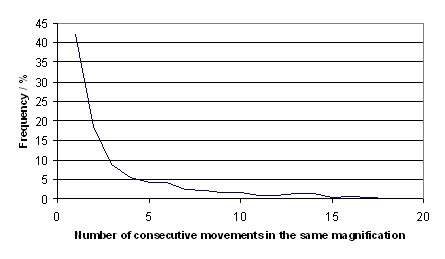
**Number of consecutive movements in the same magnification**.

In total 257 diagnostic paths from 131 WSI have been extracted and analysed. On average a diagnostic path consists of 16 image requests and takes 189 seconds between first and last image request. The results of the features which have been calculated for each path are shown in figures 5, 6, 7. The mean linearity was 0,41 and the mean 3-point-linearity 0,85.

**Figure 8:**
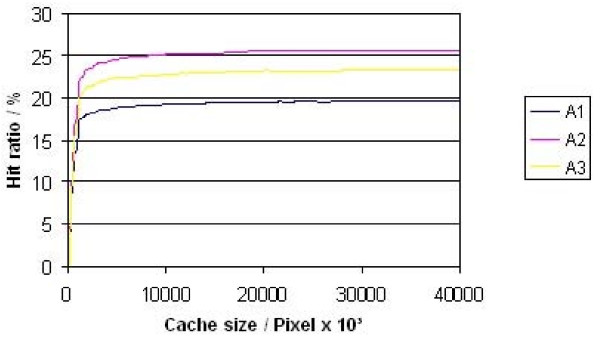
**Cache hit ratio**.

**Figure 9:**
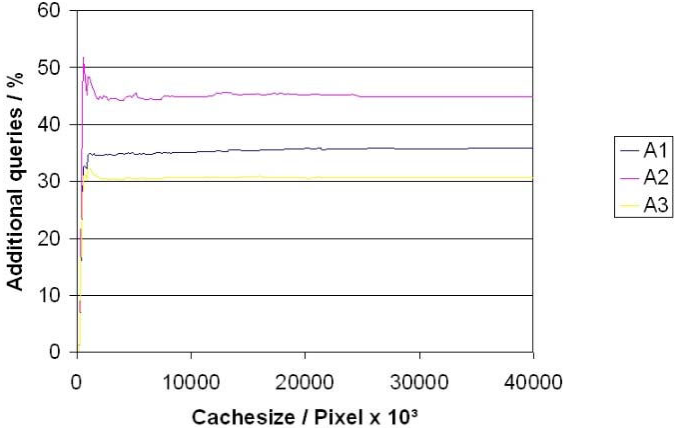
**Additional queries**.

The three different caching algorithms have been compared with respect to hit rate and additional image requests on the WSI server (fig. 8, 9). The algorithm A3 was limited to a maximum of 5 image parts for one image request. Tests demonstrated that 95% of the diagnostic paths could be loaded without any delete of entries in the cache (cache size 12,2 Megapixel). If the image parts are stored after JPEG compression this complies with less than 2 MB.

About 22% of the total image area of a diagnostic path may be loaded already by prefetching. An amount of 25,89% of the prefetched image area is needed and analysed by the pathologist. These numbers change to 21,7% and 32,4% if image parts without any image information are excluded from prefetching (fig. 8, 9).

## Discussion

WSI telepathology is a technology which offers the possibility to break the limitations of conventional static telepathology [17] and will enforce the progression in research [12,13] as well as routine pathology [14]. The complete histological slide may be investigated instead of sets of images of lesions sampled by the presenting pathologist. The benefit is demonstrated by the high diagnostic reliability of 95% accordance between first and second diagnosis. The remaining amount of 5% goes back to the diagnostic problem itself and has no correlation to the technology of WSI telepathology. Also conventional studies show a discrepancy amount of that level [16].

The characteristics of the diagnostic path for the different diagnostic procedures were not evaluated yet. We assume that there are differences due to the fact that different questions should be solved by the pathologists. In case of primary diagnosis the pathologist has to classify the tumour and to describe the in-situ-parts of the lesions as well as the surgical margin. Different views with different magnifications have to be used and the approach should be more systematically.

In the secondary and tertiary diagnostic process the first diagnosis should be confirmed by the consultant. Some diagnostic aspects are not so important now. The main question for the consultant is whether he can accept the proposed diagnosis.

We recorded some special features. Mostly short diagnostic paths have been registered. There may be two reasons for that. These are firstly the bias with respect to the study circumstances and secondly the characteristic of the diagnostic process himself, if the primary diagnosis is already known. The diagnostic process may be very fast and based on low magnifications, if in the second opinion only a carcinoma diagnosis has to be confirmed. Therefore pathologist use only one or two magnifications and move the slide systematically in few directions. That is the reason for the high values in linearity and 3-point-linearity. This underlines the possibilities of virtual microscopy where fields of view may be several times larger than under the conventional microscope. This results in a limited number of changes in position and resolution. The movements with high linearity indicate the meander like investigation of the slide as a preferred searching method in secondary diagnostic.

The possibilities to speed up the viewing process by caching and prefetching are immense. Up to 25% of the overall investigated area may be presented without long Internet dependent loading times only by use of appropriate cache memory. This value may be increased up to 33% by combination of caching and prefetching. The real possible time win depends on the characteristic of the Internet connection to the WSI server and is not simple to specify. The following figure 9 illustrates the reduction of the loading time in dependence of the Internet connection quality under the assumption that 25% of the pixels may be taken from the cache. The table was calculated for image size of 743 × 685 pixel of 66,4 Kbyte size. This image size corresponds to the working area of the used virtual microscope and a monitor resolution of 1024 × 768 pixels.

For the calculation the transfer rate was taken as net useable capacity. This is not attainable reachable in practice with respect to protocol overhead and latency periods. That means that the time savings in practical use would be higher.

## Conclusion

The general procedure of investigating a histological slide with respect to the type of diagnosis – second opinion – was in the focus of our paper. We could show that the safety of diagnostic on WSI is comparable to the conventional diagnostic based on glass slides. Discrepancies go back to problems with the difficulty of the diagnosis itself and not to technical problems with virtual microscopy.

The specific behaviour in the diagnostic process may be used for the improvement of the user acceptance of virtual microscopy. Strong reductions of loading times are possible by using caching and prefetching.

Within further investigations we will analyse the user behaviour for other diagnostic procedures (primary and tertiary diagnostic) based on the investigation of diagnostic paths. Our goal is the development of special strategies for caching and prefetching with respect to the type of diagnosis and the diagnostic question.

## Competing interests

The authors declare that they have no competing interests.

## Authors' contributions

SW developed the algorithms and evaluated them. MB participated in the design of the study and evaluated the algorithms. KS developed the WSI interface and the server technology. PH designed the telepathology study and drafted the manuscript. TS designed and carried out the telepathology study and drafted the manuscript. All authors read and approved the final manuscript.
